# Circulating miR-485-5p as a potential diagnostic and prognostic biomarker for HCV-related hepatocellular carcinoma

**DOI:** 10.1007/s10238-025-01625-y

**Published:** 2025-04-10

**Authors:** Gamalat A. Elgedawy, Naglaa S. Elabd, Asmaa M. Elbrolosy, Suzan M. El-Morshedy, Ayman El-Gamal, Mai Abozeid, Mervat Abdelkreem, Sama S. Eleowa, Marwa L. Helal

**Affiliations:** 1 Clinical Biochemistry and Molecular Diagnostics Department, National Liver Institute, Menoufia University, Shebin El-Kom, Menoufia Egypt; 2 Tropical Medicine Depatment, Faculty of Medicine, Menoufia University, Shebin El-Kom, Menoufia 32511 Egypt; 3 Medical Microbiology and Immunology Department, Faculty of Medicine, Menoufia University, Shebin El-Kom, Menoufia Egypt; 4 Clinical Pathology Department, National Liver Institute, Menoufia University, Shebin El-Kom, Menoufia Egypt; 5 Hepatology and Gastroenterology Department, National Liver Institute, Menoufia University, Shebin El-Kom, Menoufia 32511 Egypt; 6 BMS-University of Science and Technology at Zewail City, Giza, Egypt

**Keywords:** HCV, Hepatocellular carcinoma, Biomarker, MiR-485-5p

## Abstract

Hepatitis C virus (HCV) is the predominant viral cause of hepatocellular carcinoma (HCC). Early detection and use of reliable biological markers can improve survival for HCC patients. MiR-485-5p was identified as a tumor-suppressing microribonucleic acid (miRNA) in some human cancers and was recently found to be downregulated in HCC tissues, signifying its utility as a promising biomarker. We aimed to investigate the potential role of circulating miR-485-5p as a novel diagnostic and prognostic biomarker for HCV-related HCC. This case–control study included 50 patients with HCC associated with HCV, 50 patients with HCV-related liver cirrhosis, and 50 healthy controls. History gathering, physical examination, laboratory, and imaging assessments were performed. A quantitative real-time polymerase chain reaction was used to measure miR-485-5p levels. Serum miR-485-5p values demonstrated a stepwise decline pattern from the control group to cirrhotic patients, with the HCC group exhibiting the lowest levels (*p* < 0.001). HCC patients with early BCLC stages had significantly lower miR-485-5p levels than those with late stages (*p* = 0.004). The miR-485-5p displayed a better performance in predicting HCV-related HCC with a greater area under the ROC curve (AUC) than alpha-fetoprotein (AFP) (AUC and sensitivity 0.921 and 92.0 versus 0.704 and 64.0, respectively) (*p* < 0.001). Also, its performance in predicting HCC prognosis surpassed that of AFP (AUC and sensitivity 0.872 and 85.19 versus 0.695 and 62.96, respectively) (*p* < 0.001). Circulating miR-485-5p is a promising, accurate, and noninvasive biomarker for the early detection and prediction of prognosis in patients with HCV-linked HCC.

**Supplementary Information:**

The online version contains supplementary material available at 10.1007/s10238-025-01625-y.

## Background

Liver cancer accounts for 4.7% of newly diagnosed cases and 8.3% of all cancer-related deaths. It is the sixth most frequently diagnosed cancer and the third leading cause of cancer-related death [1, 2]. Hepatocellular carcinoma (HCC) is the most prevalent of all types of liver cancer. It is responsible for over 75% of primary liver cancer cases globally [3]. The considerable variations in HCC incidence and mortality rates may be attributed to epigenetic and genetic alterations, differences in environmental exposure, timing, and chronic viral hepatitis prevalence, as well as differences in the availability of medical care and the ability to identify and treat HCC at an early stage [4]. HCV is the etiology most frequently associated with HCC. Nearly all patients with HCV-related HCC have cirrhosis at the time of diagnosis. Numerous direct interactions between viral components and cellular tumor suppressors or oncogenes were found in HCV molecular virology research. HCV directly stimulates Wnt/β-catenin signaling in infected cells, and clinical data suggest that this effect prevails even after viral clearance due to epigenetic alterations [5].

HCC is often diagnosed, typically at an advanced stage, only when symptoms become apparent. It is well established that the 5-year survival rate of HCC is closely correlated with the disease stage [6]. Using a reliable biomarker is crucial for improving the early detection of HCC. Alpha-fetoprotein (AFP) is the most widely used for the diagnosis of HCC. It has low specificity and sensitivity, which limits its clinical utility [7]. Early-stage HCC may not elevate AFP levels. Normal AFP values were found in some patients with advanced-stage HCC. Furthermore, it may rise in some patients with liver cirrhosis, chronic hepatitis, and other liver diseases, leading to high false-positive rates [8].

Functional noncoding ribonucleic acids (RNAs) include ribosomal RNA, transfer RNA, and small nuclear ribonucleic acids that process pre-mRNA, long noncoding RNA (lncRNA), as well as microribonucleic acids (miRNAs) [9, 10]. It was found that miRNAs have extraordinary roles in coordinating many cellular biological processes, including apoptosis, differentiation, and proliferation. This function is associated with miRNAs' capacity to attach to particular target mRNA sites at the 3′UTR, which may result in mRNA degradation or translational repression [11]. HCV influences the expression of miRNAs in hepatocytes, promoting tumorigenesis. Furthermore, miRNA dysregulation has been associated with chronic hepatitis C initiation and progression to liver cirrhosis and HCC [12, 13]. Over 100 miRNAs have been discovered to be dysregulated in HCC tumor tissue compared to nontumor tissue. However, the plasma of HCC cases showed aberrant expression of only a small fraction of circulating miRNAs [14].

It was reported that miR-485-5p exhibited a tumor suppressor role in a range of malignancies, and it has been observed that several cancer cells have downregulated miR-485-5p [15–20]. Likewise, miR-485-5p was significantly downregulated in human HCC tissues, and it was recently found to inhibit HCC progression by disrupting the WBP2/Wnt signaling pathway [21–23]. Several studies suggested miR-485-5p as a common target of different lncRNAs that promote HCC progression via sponging miR-485-5p [24–26]. Circulating miR-485-5p has shown promise as a potential biomarker for some cancers [27, 28]. Evidence about its clinical significance as a potential biomarker in HCC patients is lacking. The available data collectively indicate that miR-485-5p holds promise as a potential biomarker for diagnosing as well as assessing the stage of HCC and hence patients' prognosis. In this regard, we aimed to investigate the potential role of miR-485-5p in HCC diagnosis and study its relations with clinical, biochemical, and radiological characteristics, as well as the prognosis of patients with HCC.

## Methods

This case–control study was conducted on 150 participants, recruited from the inpatient wards and the outpatient clinics of the National Liver Institute, Menoufia University, Egypt, and the Tropical Medicine Department, Faculty of Medicine, Menoufia University, Egypt, between March 2023 and March 2024. These subjects were categorized into three groups: 50 patients with HCV-related HCC, 50 patients with HCV-related liver cirrhosis without HCC, and 50 healthy controls of comparable age and gender. To achieve a statistical power of 80% (0.8), the sample size was calculated by using the OpenEpi website (http://www.openepi.com/SampleSize/SSCC.htm).

All participants were subjected to a thorough history gathering and physical examination. Laboratory investigations included a complete blood count, renal function tests, liver function tests, AFP, serological tests for viral markers by enzyme-linked immunosorbent assay (ELISA), including HBsAg, HCV, and HIV antibodies, and real-time PCR for HCV. Radiological evaluation was done initially through a pelvic-abdominal ultrasound. Liver cirrhosis diagnosis was determined by clinical, laboratory, and ultrasonography assessments. Patients with suspected HCC underwent a triphasic CT scan of the chest, abdomen, and pelvis for confirming HCC diagnosis and determining the presence of distant metastases. The diagnosis of HCC was based on the characteristic triphasic CT findings of arterial-phase enhancement and portal/delayed washout [29].

All patients (both the liver cirrhosis and HCC groups) had a history of HCV treatment with direct-acting antivirals (DAAs). They were enrolled during their follow-up visits after anti-HCV treatment. Patients with HCC were enrolled before starting any line of treatment for HCC.

Patients with liver diseases other than HCV, such as chronic Hepatitis B virus, autoimmune hepatitis, metabolic hepatitis, diabetes mellitus, metabolic syndrome, and a history of alcohol or hepatotoxic drug use, as well as patients with hepatic focal lesions other than HCC, were excluded.

Additionally, in cirrhotic as well as HCC patients, the Child–Turcotte–Pugh score was calculated to assess the severity of liver disease [30]. Esophagogastroduodenoscopy was performed for the identification and grading of gastroesophageal varices.

According to the most recent official update of the Barcelona clinic liver cancer (BCLC) 2022 [31], patients with HCC were divided into two subgroups based on their expected survival: HCC-subgroup I (Early BCLC), which included patients with expected survival more than 5 years involving BCLC 0 and BCLC-A, and HCC-subgroup II (Late BCLC), which included patients with expected survival less than five years involving BCLC-B, BCLC-C, and BCLC-D.

### Biochemical investigations

The obtained 10 ml of blood from venipuncture was divided into 3 parts. The first 5 ml was placed into a plain tube, centrifuged, and the produced sera were used for all biochemical tests conducted on (SYNCHRON CX9ALX, Beckman, CA, USA) and for AFP and HCV RNA, which were measured on (Architect i1000SR, Abbott, USA). The second 2 ml was used to measure the INR, and the third 3 ml was withdrawn into two EDTA tubes; one was used for immediate CBC counting, which was performed on an automated Sysmex KX-21 (Sysmex Corporation, Kobe, Japan), and the second was used for miRNA 485-5P extraction.

### MiR-485-5p isolation

MiRNA was isolated using the producer’s protocol by miRNeasy Mini Kit (Qiagen, Hilden, Germany) using 200 μL of fresh blood samples. NanoDrop 2000 spectrophotometry was used to quantify the yield of miRNA (Thermo Scientific, Wilmington, Delaware, USA). For cDNA synthesis procedure: mixing reverse transcription (RT) primer (3 μL), 10 RT buffer (1.5 μL), reverse transcriptase (1 μL), RNase inhibitor (0.19 μL), deoxynucleotide (dNTP) (0.15 μL), and nuclease-free water (4.16 μL) (using TaqMan miRNA Reverse Transcription Kit, Applied Biosystems, Foster City) with (5 μL) of RNA sample. Thermal cycler conditions: 16 °C for 30 min, 42 °C for 30 min, and 85 °C for 5 min using ProFlex, Applied Biosystems, Thermo Fisher Scientific.

For miR-485-5p expression, a 20-μL reaction volume was prepared containing 3 μL of RNase-free water, 10 μL of SYBER Green universal master mix, 1 μL of each primer, and 5 μL of prepared cDNA. Cycling conditions: an initial denaturation of 95 °C for 15 min, then another 45 cycles of denaturation at 95 °C for 15 s, annealing, and extension at 60 °C for 60 secs (using the 7500 fast real-time instrument, Applied Biosystems, Thermo Fisher Scientific).

Gene's primers (Qiagen, Hilden, Germany) were:

- MiR-485-5p forward primer: 5'-GGAGAGGCTGGCCGTGAT-3'.
- MiR-485-5p reverse primer: 5ʹ-CAGTGCGTGTCGTGGAGT-3ʹ.
- U6 snRNA forward primer: 5'-GCTTCGGCAG CACATATACTAAAAT-3'.
- U6 snRNA reverse primer: 5ʹ-CAGTGCGTGTCGTGGAGT-3ʹ.

Comparing the target gene expression to the reference-control gene (normalized to the expression of U6 snRNA): relative expression = 2-ΔΔCT, where CT is the cycle threshold for every sample and ΔΔCT = ΔCT (tested sample) − ΔCt (control).

ΔCT = CT (a target gene)–CT (a reference gene).

ΔΔCT = ΔCT (a target sample)–ΔCT (a control sample).

### Statistical analysis

Data were analyzed by utilizing IBM SPSS software package version 20.0. (Armonk, NY: IBM Corp). Categorical data were described in numbers and percentages. The chi-square test was applied to compare between two groups. The continuous data were tested for normality by the Shapiro–Wilk test. Quantitative data were displayed as range (minimum and maximum), mean, standard deviation, and median. The F-test (ANOVA) was used for comparing the three studied groups for normally distributed quantitative variables, followed by the post hoc test (Tukey) for pairwise comparison. The Kruskal–Wallis test was used to compare different groups for not normally distributed quantitative variables, followed by the post hoc test (Dunn's for multiple comparisons test) for pairwise comparison. The Mann–Whitney test was applied to compare two groups for abnormally distributed quantitative variables. The Spearman coefficient was used to correlate between quantitative variables. The receiver operating characteristic curve (ROC) was utilized to determine the diagnostic performance of the markers. Significance was judged at the 5% level.

## Results

**Fig. 1:**
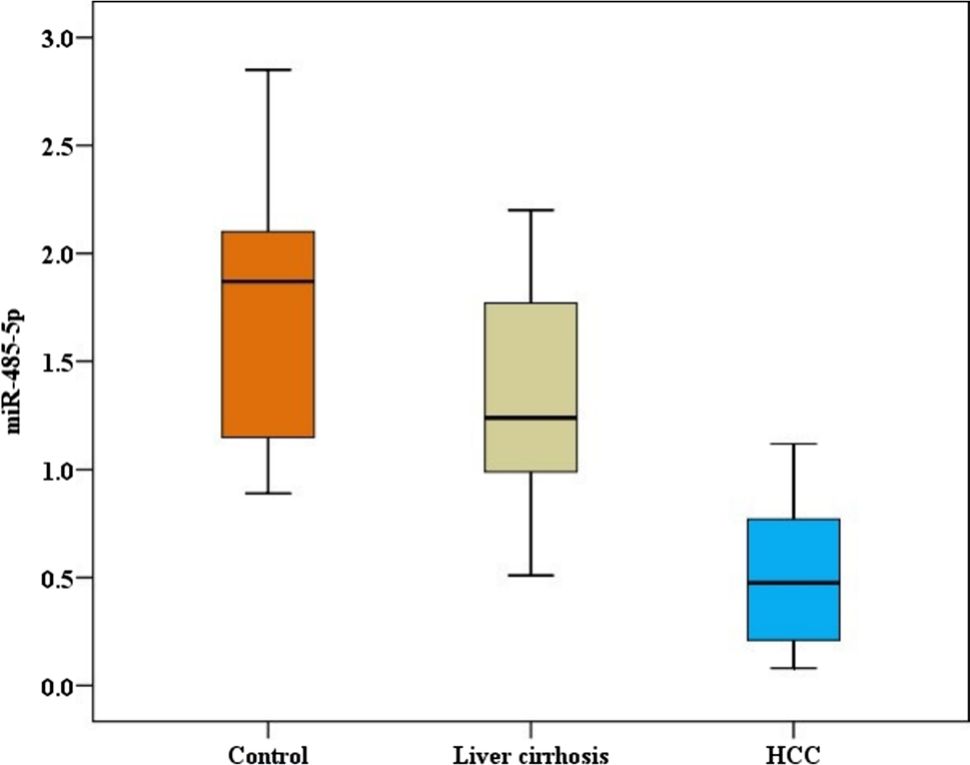
miR-485-5p levels among studied groups

Table 1 summarizes the demographic, clinical, and imaging characteristics of the studied groups. The laboratory parameters of the studied groups are illustrated in Table 2. Patients with HCC had significantly higher AFP levels than cirrhotic patients and controls. The cirrhotic group also had significantly higher levels of AFP than controls (*P* < 0.001). The miR-485-5p values demonstrated a stepwise decline pattern from the control group to cirrhotic patients, with the HCC group exhibiting the lowest levels (*p* < 0.001) (Table 3, Fig. 1).

**Table 1:** Demographic, clinical, and imaging characteristics of the studied groups

		Control (n = 50)	Liver cirrhosis (n = 50)	HCC (n = 50)	Test	*p*
Sex	Male	26 (52.0%)	28 (56.0%)	31 (62.0%)	χ^2^ = 1.032	0.597
	Female	24 (48.0%)	22 (44.0%)	19 (38.0%)		
Age	Mean ± SD	49.4 ± 5.1	50.3 ± 4.6	51.3 ± 4.6	F = 1.938	0.148
	Median (Min.–Max.)	49.5(40–57)	50 (42–59)	51 (43–64)		
History of anorexia		–	9 (18.0%)	16 (32.0%)	χ^2^ = 2.613	0.106
History of abdominal pain		–	10 (20.0%)	15 (30.0%)	χ^2^ = 1.333	0.248
History of Encephalopathy		–	13 (26.0%)	21 (42.0%)	χ^2^ = 2.852	0.091
History of weight loss		–	7 (14.0%)	17 (34.0%)	χ^2^ = 5.482	0.019*
Edema lower limbs		–	21 (42.0%)	17 (34.0%)	χ^2^ = 0.679	0.410
Jaundice		–	13 (26.0%)	14 (28.0%)	χ^2^ = 0.051	0.822
Ascites		–	31 (62.0%)	23 (46.0%)	χ^2^ = 2.576	0.108
Child–Pugh class	A	–	17 (34.0%)	19 (38.0%)	χ^2^ = 1.112	0.573
	B	–	22 (44.0%)	17 (34.0%)		
	C	–	11 (22.0%)	14 (28.0%)		
Gastroesophageal Varices		_	23 (46.0%)	26 (52.0)	χ^2^ = 0.3601	0.548
CT findings						
Liver size	Average size	–	6 (12.0%)	4 (8.0%)	χ^2^ = 6.4533	0.396
	Hepatomegaly	–	3 (6.0%)	12 (24.0%)		
	Shrunken liver	_	41 (82.0%)	34 (68.0%)		
Portal vein diameter	Normal	–	18 (36.0%)	23 (46.0%)	χ^2^ = 1.0335	0.309
	Dilated	–	32 (64.9%)	27 (54.0%)		
Portal vein patency	Patent	–	50 (100.0%)	44 (88.0%)	–	–
	Thrombosed	–	0 (0.0%)	4 (8.0%)		
	Malignant invasion	–	0 (0.0%)	2 (4.0%)		
Spleen size	Average size	_	13 (26.0%)	10 (20%)	*χ*^2^ = 0.5082	0.475
	Splenomegaly	_	37 (74.0%)	40 (80%)		
Number of focal lesions	Single	_	_	33 (66%)	–	–
	Multiple	_	_	17 (34%)		
Size of focal lesions (cm)	Mean ± SD	_	_	4.6 ± 1.6	–	–
	Median (Min.–Max.)	_	_	4.9 (1.4–7.7)		
BCLC staging	HCC-subgroup I (BCLC 0 and A)	_	_	23(46.0%)	–	–
	HCC-subgroup II (BCLC-B, C, and D)	_	_	27(54.0%)		

SD: Standard deviation, Min.: Minimum, Max.: Maximum, n: Number, χ^2^: Chi-square test, F: F for ANOVA test, *p*: *p* value, HCC: hepatocellular carcinoma, BCLC: Barcelona clinic liver cancer. *Statistically significant

**Table 2:** Laboratory characteristics of the studied groups

		Control (n = 50)	Liver cirrhosis (n = 50)	HCC (n = 50)	Test	*p*
Hemoglobin (gm/dl)	Mean ± SD	13.3 ± 1.3	10.2 ± 1.4	11.5 ± 1.9	F = 52.235	< 0.001*
	Median (Min.–Max.)	13 (11.3–16.3)	10.1 (8–14.3)	11.5 (7.3–15.6)		
		*p*_1_ < 0.001*, *p*_2_ < 0.001*, *p*_3_ < 0.001*				
WBCs (× 1000/ul)	Mean ± SD	6.9 ± 1.46	6.6 ± 1.8	6.1 ± 2.2	H = 4.149	0.126
	Median (Min.–Max.)	6.7 (4.3–10.1)	6.6 (2.2–9.2)	5.9(2.3–9.6)		
Platelets (× 1000/ul)	Mean ± SD	272.6 ± 49.4	113.9 ± 52.6	127.7 ± 76.6	H = 80.938	< 0.001*
	Median (Min.–Max.)	266 (184–360)	104 (41–314)	106 (39–366)		
		*p*_1_ < 0.001*, *p*_2_ < 0.001*, *p*_3_ = 0.574				
RBS (mg/dl)	Mean ± SD	95.4 ± 12	102.9 ± 16.8	106.6 ± 14.1	F = 7.848	0.001*
	Median (Min.–Max.)	96 (71–120.4)	105 (73–136.6)	105.8 (79.5–135)		
		*p*_1_ = 0.027*, *p*_2_ < 0.001*, *p*_3_ = 0.411				
Creatinine (mg/dl)	Mean ± SD	0.8 ± 0.2	1 ± 0.3	1 ± 0.3	F = 11.328	< 0.001*
	Median (Min.–Max.)	0.8 (0.5–1.2)	1 (0.4–1.7)	1 (0.6–1.5)		
		p_1_ = 0.001*, p_2_ < 0.001*, p_3_ = 0.633				
AST	Mean ± SD	19.1 ± 3.7	58 ± 23.3	72.6 ± 30.6	H = 98.864	< 0.001*
	Median (Min.–Max.)	19 (11–27)	51 (20–111)	67 (24–134)		
		*p*_1_ < 0.001*, *p*_2_ < 0.001*, *p*_3_ = 0.098				
ALT	Mean ± SD	18.2 ± 6.5	47.8 ± 22.2	68.7 ± 32.7	H = 99.181	< 0.001*
	Median (Min.–Max.)	17 (9–36)	41 (19–104)	56 (34–161)		
		*p*_1_ < 0.001*, *p*_2_ < 0.001*, *p*_3_ = 0.003*				
ALP	Mean ± SD	61 ± 13	100.1 ± 30.4	121.2 ± 40.8	F = 50.676	< 0.001*
	Median (Min.–Max.)	62.4 (33–89.8)	97.5 (46–184)	110 (50–202)		
		*p*_1_ < 0.001, *p*_2_ < 0.001, *p*_3_ = 0.002				
GGT	Mean ± SD	20.8 ± 8.5	43.7 ± 21.6	83.6 ± 45.5	H = 75.931	< 0.001*
	Median (Min.–Max.)	20 (8.8–41)	38 (18–100)	83 (18–177)		
		*p*_1_ < 0.001*, *p*_2_ < 0.001*, *p*_3_ = 0.001*				
Total bilirubin	Mean ± SD	0.5 ± 0.2	2.1 ± 1	2.1 ± 1.7	H = 93.766	< 0.001*
	Median (Min.–Max.)	0.5 (0.1–0.8)	1.8 (1.1–4.9)	1.4 (0.4–7.5)		
		*p*_1_ < 0.001*, *p*_2_ < 0.001*, *p*_3_ = 0.145				
Direct bilirubin	Mean ± SD	0.1 ± 0	1 ± 0.6	1.1 ± 1.2	H = 93.612	< 0.001*
	Median (Min.–Max.)	0.1 (0.1–0.2)	0.8 (0.4–2.6)	0.5 (0.1–4.9)		
		*p*_1_ < 0.001*, *p*_2_ < 0.001*, *p*_3_ = 0.054				
Albumin	Mean ± SD	4.3 ± 0.4	2.9 ± 0.7	2.9 ± 0.8	F = 80.214	< 0.001*
	Median (Min.–Max.)	4.3 (3.5–5.1)	2.9 (1.5–4.3)	3 (1.3–4.5)		
		*p*_1_ < 0.001*, *p*_2_ < 0.001*, *p*_3_ = 0.797				
INR	Mean ± SD	1 ± 0	1.4 ± 0.3	1.4 ± 0.4	H = 87.218	< 0.001*
	Median (Min.–Max.)	1 (1–1.1)	1.3 (1–2.2)	1.3 (1–3)		
		*p*_1_ < 0.001*, *p*_2_ < 0.001*, *p*_3_ = 0.712				

HCC: hepatocellular carcinoma, n: Number, WBCs: White blood cells, RBS: Random blood sugar, AST: Aspartate transaminase, ALT: Alanine transaminase, ALP: Alkaline phosphatase, GGT: Gamma-glutamyl transferase, INR: International normalized ratio, SD: Standard deviation, Min.: Minimum, Max.: Maximum, F: F for ANOVA test, H: H for Kruskal–Wallis test, *p*: *p* value, *p*_1_: control vs. liver cirrhosis, *p*_2_: control vs. HCC, *p*_3_: liver cirrhosis vs. HCC. *Statistically significant

**Table 3:** Comparison between the studied groups according to AFP and miR-485-5p

	Control (n = 50)	Liver cirrhosis (n = 50)	HCC (n = 50)	H	*p*
AFP ng/mL					
Mean ± SD	3.5 ± 1.9	40.6 ± 35.41	755.1 ± 1585.4	83.571	< 0.001*
Median (Min.–Max.)	3.3 (0.8–6.9)	30 (2–135)	68 (2.4–7150)		
	*p*_1_ < 0.001*, *p*_2_ < 0.001*, *p*_3_ = 0.008*				
miR-485-5p					
Mean ± SD	1.7 ± 0.50	1.30 ± 0.49	0.5 ± 0.3	83.899	< 0.001*
Median (Min.–Max.)	1.9(0.9–2.9)	1.24 (0.51–2.20)	0.5 (0.1–1.1)		
	*p*1 = 0.002*, *p*_2_ < 0.001*, *p*_3_ < 0.001*				

HCC: hepatocellular carcinoma, n: Number, AFP: alpha-fetoprotein, SD: Standard deviation, Min.: Minimum, Max.: Maximum, H: H for Kruskal–Wallis test, *p*: *p* value, *p*_1_: control vs. liver cirrhosis, *p*_2_: control vs. HCC, *p*_3_: liver cirrhosis vs. HCC. *Statistically significant

**Fig. 2:**
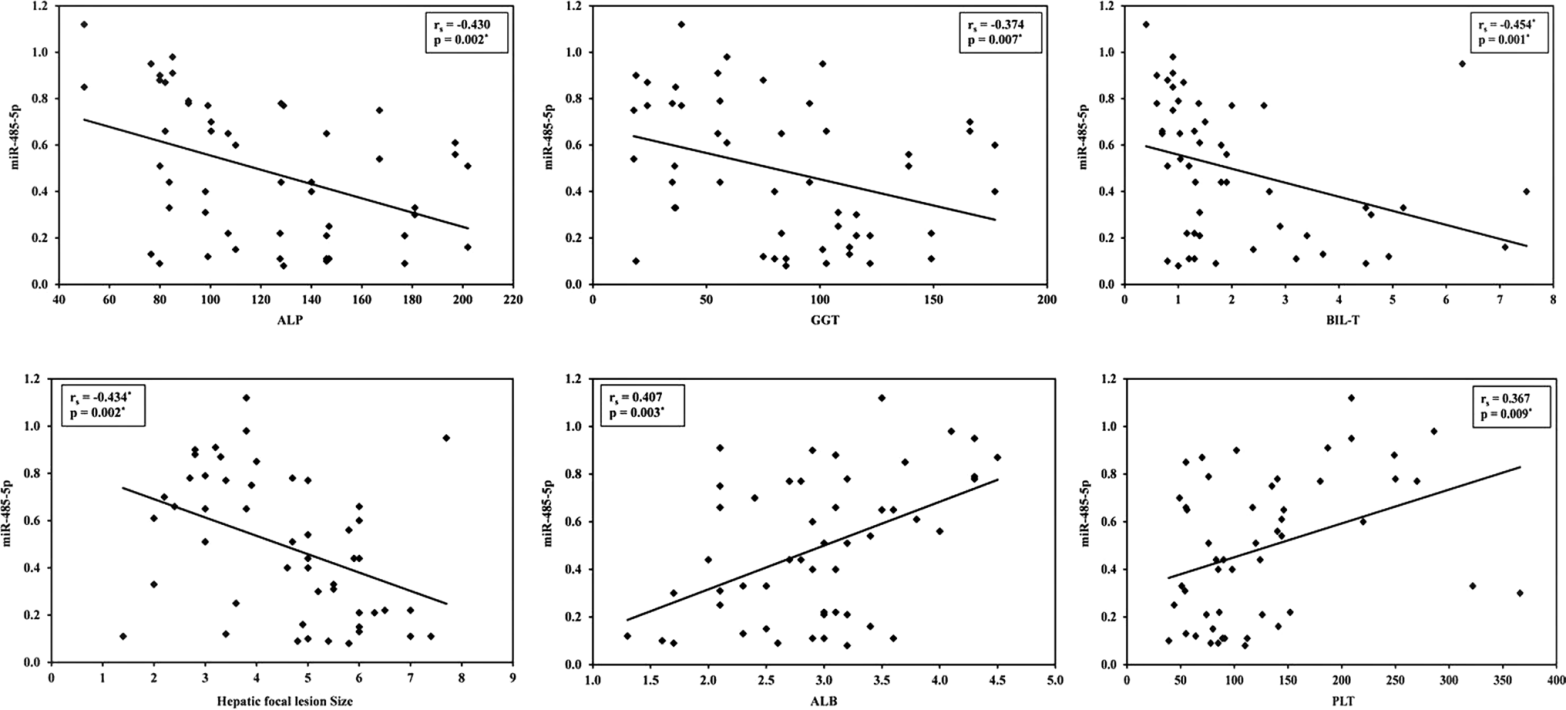
Correlation between miR-485-5p and different parameters in HCC group. ALP: Alkaline phosphatase, GGT: Gamma-glutamyl transferase, BIL-T: Total bilirubin, ALB: Albumin, PLT: Platelets, rs: Spearman coefficient, *p*: *p* value.

The levels of AFP and miR-485-5p in the HCC patient group showed a significant difference with the Child–Turcotte–Pugh class (*p* = 0.014 and < 0.001, respectively). In contrast to AFP, the miR-485-5p values showed a progressive decrease from class A to class B to class C. Lower miR-485-5p levels were significantly associated with the existence of multiple hepatic focal lesions (Table 4). AFP levels were negatively correlated with miR-485-5p (*p* < 0.001). Furthermore, miR-485-5p values showed a significant negative correlation with alkaline phosphatase (ALP), gamma-glutamyl transferase (GGT), total bilirubin, and the size of the hepatic focal lesions and a significant positive correlation with albumin and platelet count (Supplementary Table 1, Fig. 2).

**Table 4:** Relation between AFP and miR-485-5p with different parameters in HCC patients (n = 50)

	No	AFP ng/mL		miR-485-5p	
		Mean ± SD	Median (Min.–Max.)	Mean ± SD	Median (Min.–Max.)
Sex					
Male	31	625.3 ± 1482.9	45.63 (2.40–7150.0)	0.53 ± 0.28	0.60 (0.08–0.98)
Female	19	966.9 ± 1760.9	240.0 (5.50–7150.0)	0.42 ± 0.32	0.33 (0.09–1.12)
U (p)		234.000 (0.226)		222.500 (0.150)	
Child–Pugh class					
A	19	658.7 ± 1715.8	68.0 (2.40–7150.0)	0.55 ± 0.28	0.61 (0.10–0.91)
B	17	459.2 ± 970.3	50.0 (5.50–3920.0)	0.46 ± 0.26	0.40 (0.13–0.98)
C	14	1245.4 ± 1962.5	417.0 (2.40–7150.0)	0.44 ± 0.37	0.32 (0.08–1.12)
H (p)		6.047 (0.014*)		19.042 (< 0.001*)	
Hepatic focal lesion number					
Single	33	677.6 ± 1785.8	45.63 (2.40–7150.0)	0.62 ± 0.27	0.65 (0.10–1.12)
Multiple	17	905.7 ± 1132.5	455.0 (10.14–3920.0)	0.23 ± 0.16	0.21 (0.08–0.66)
U (p)		160.500 (0.014*)		67.500 (< 0.001*)	

AFP: alpha-fetoprotein, SD: Standard deviation, Min.: Minimum, Max.: Maximum, U: Mann–Whitney test, H: H for Kruskal–Wallis test, *p*: *p* value**.** *Statistically significant

**Fig. 3:**
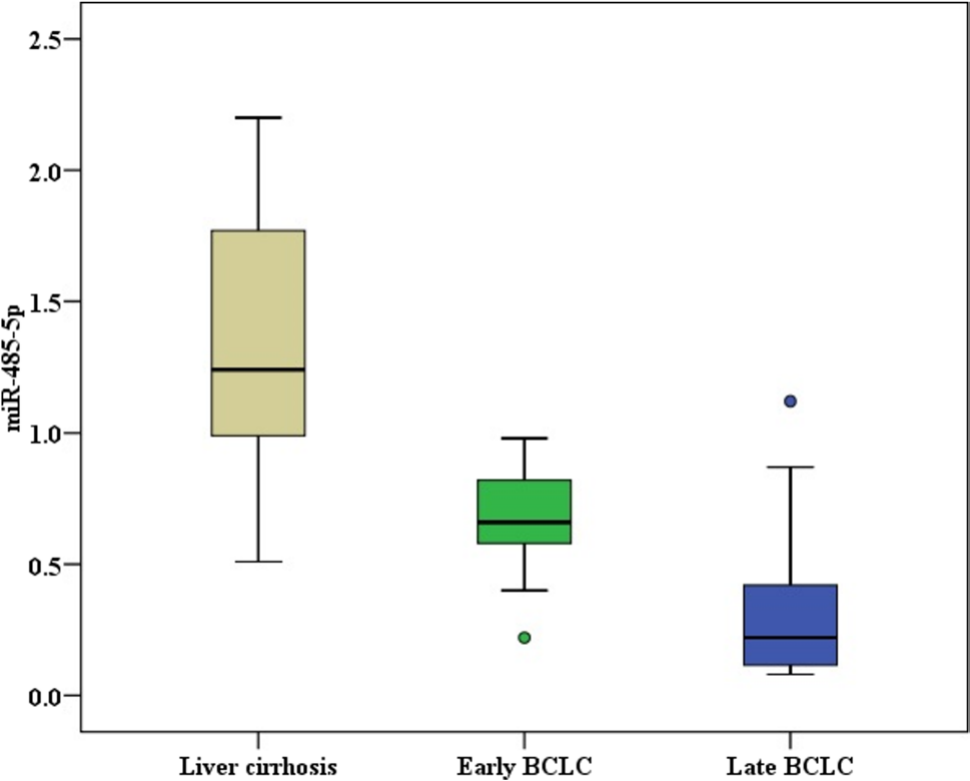
miR-485-5p levels among liver cirrhosis group and HCC subgroups

HCC-subgroup I (early BCLC) included 23 patients with BCLC 0 or A. HCC-subgroup II (late BCLC) included 27 patients with BCLC-B, C, or D. The miR-485-5p values were significantly lower in HCC-subgroup I and HCC-subgroup II compared to the liver cirrhosis group (*p* < 0.001). In addition, HCC-subgroup II had lower values than HCC-subgroup I (*p* = 0.004) (Fig. 3). AFP values were significantly higher in HCC-subgroup I than HCC-subgroup II, but it showed no significant difference between the liver cirrhosis group and HCC-subgroup I (Table 5).

**Table 5:** Comparison between liver cirrhosis, early BCLC, and late BCLC groups according to AFP and miR-485-5p

	Liver cirrhosis (n = 50)	HCC-subgroup I [Early BCLC] (n = 23)	HCC-subgroup II [Late BCLC] (n = 27)	H	*p*
AFP ng/mL					
Mean ± SD	40.6 ± 35.41	192.1 ± 356.3	1234.8 ± 2027.3	17.566	< 0.001*
Median (Min.–Max.)	30 (2–135)	40.6 (2.4–1360)	240 (10.14–7150)		
	*p*_1_ = 0.164, *p*_2_ < 0.001*, *p*_3_ = 0.022*				
miR-485-5p					
Mean ± SD	1.30 ± 0.49	0.69 ± 0.19	0.32 ± 0.27	60.758	< 0.001*
Median (Min.–Max.)	1.24 (0.51–2.20)	0.66 (0.22–0.98)	0.22 (0.08–1.12)		
	*p*_1_ < 0.001*, *p*_2_ < 0.001*, *p*_3_ = 0.004*				

HCC: hepatocellular carcinoma, BCLC: Barcelona clinic liver cancer, n: Number, AFP: alpha-fetoprotein, SD: Standard deviation, Min.: Minimum, Max.: Maximum, H: H for Kruskal–Wallis test, *p*: *p* value, *p*_1_: liver cirrhosis vs. early BCLC, *p*_2_: liver cirrhosis vs. late BCLC, p_3_: early BCLC vs. late BCLC. *Statistically significant

**Fig. 4:**
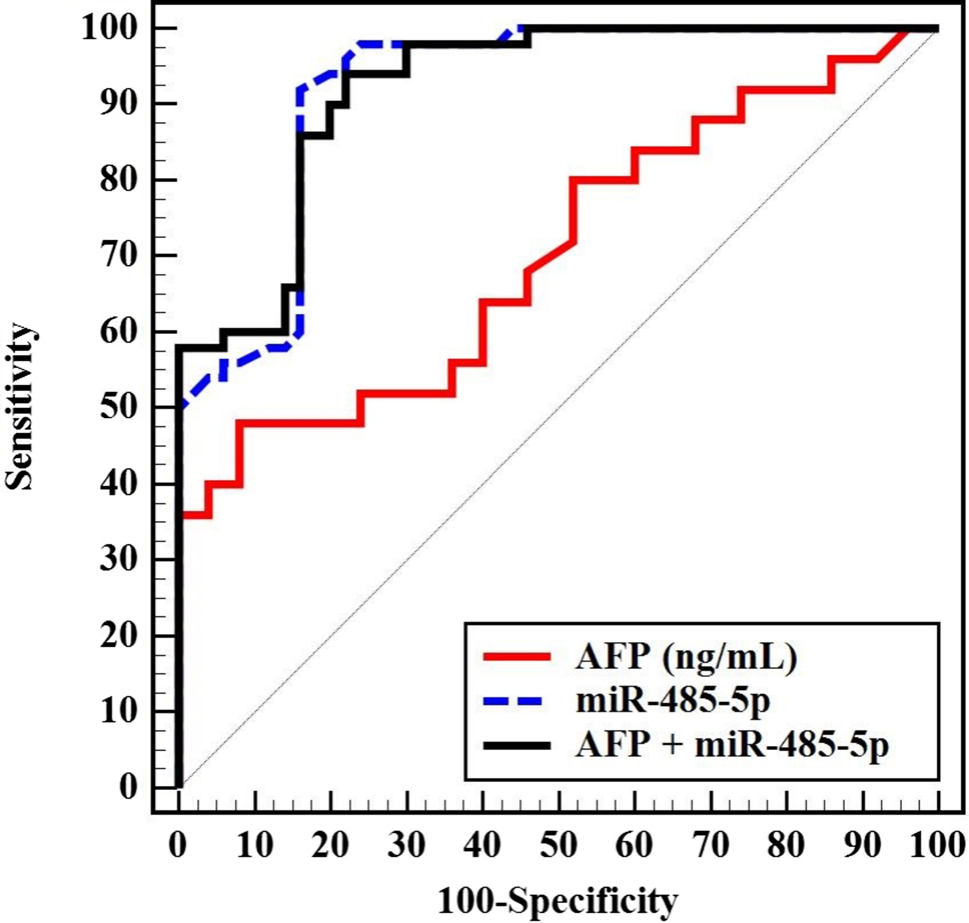
ROC curve for the diagnostic performance of miR-485-5p

**Fig. 5:**
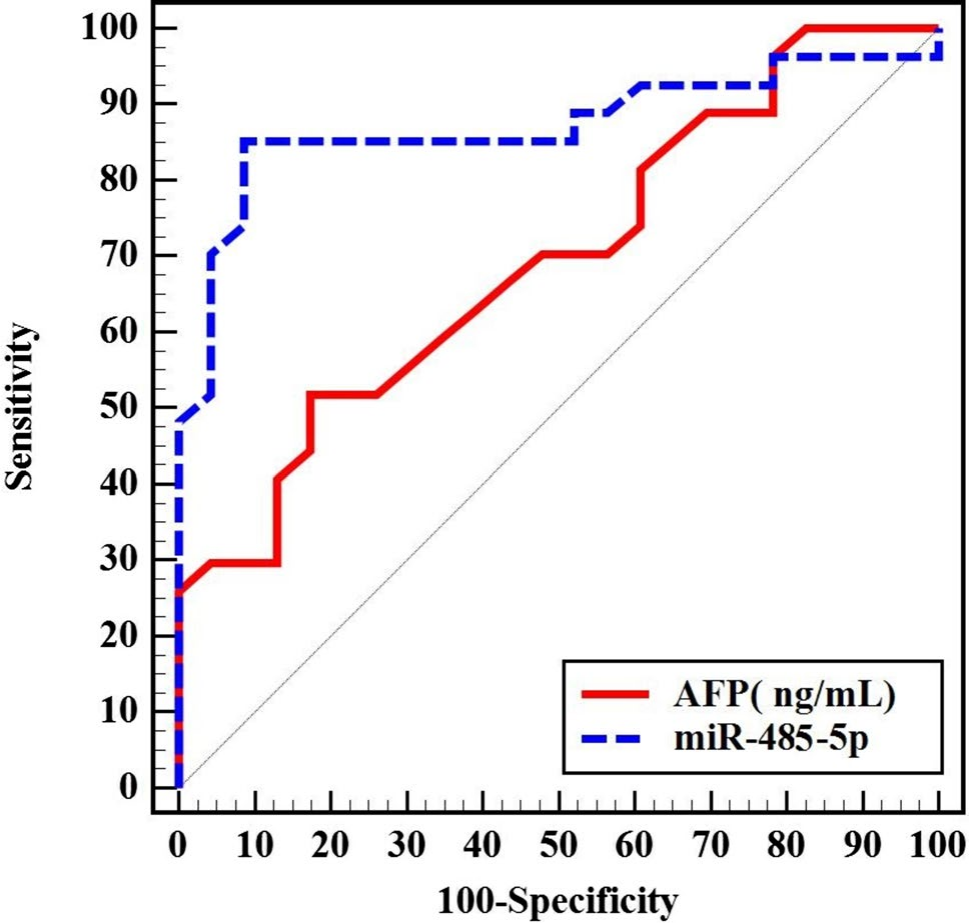
ROC curve for the prognostic performance of miR-485-5p

Both AFP and miR-485-5p were able to discriminate HCC patients from those with liver cirrhosis (*p* < 0.001). The miR-485-5p displayed a better performance with greater area under the ROC curve (AUC) and a higher sensitivity and specificity than AFP in predicting HCC in HCV-related liver cirrhosis (AUC, sensitivity, and specificity of 0.921, 92.0, and 84.0 for miR-485-5p, and 0.704, 64.0, and 60.0 for AFP, respectively) (Fig. 4). The miR-485-5p outperformed AFP in predicting prognosis in HCC patients by distinguishing between early and late stages of BCLC, with higher AUC, sensitivity, and specificity (0.872, 85.19, and 82.61 versus 0.695, 62.96, and 60.87, respectively) (Fig. 5) (Supplementary Table 2).

## Discussion

HCC has always posed a major threat to global health and is a serious public health concern in Egypt. The government's mass screening program for HCV identification and management may be a contributing factor to the increased rates of HCC detection in Egypt [32]. HCV infection is a major risk factor for liver cirrhosis and is associated with a 20-fold increase in HCC development risk. In addition, 0.5–10% of cirrhotic patients are at risk of HCC annually [33]. Due to the development of interferon (IFN)-free, direct-acting antiviral (DAA) therapy, over 95% of patients treated for HCV infection can now achieve a sustained viral response (SVR) safely and in a short amount of time, regardless of their underlying liver conditions. Although achieving SVR is the goal of HCV treatment, the risk of developing HCC remains high even after HCV elimination, particularly in patients with advanced fibrosis and cirrhosis. HCV-related HCC is primarily caused by HCV protein expression in infected hepatocytes, repeated inflammation, damage, and regeneration with the accumulation of epigenetic and genetic alterations and malignant transformations [34].

Improving patient outcomes requires early detection of HCC. Currently, though, there is disagreement over the best screening technique for HCC. Ultrasound is the most popular and reasonably priced screening method for early HCC detection in high-risk groups, but it has its limitations when it comes to sensitivity and accuracy [35]. A number of biomarkers have been studied as prognostic and diagnostic markers for HCC, including AFP, glypican-3, and des-gamma-carboxy prothrombin. Newer biomarkers and imaging methods are being investigated to enhance HCC early diagnosis. Recent research studies have examined long noncoding RNAs and miRNAs for more precise and early HCC detection [36].

Circulating miR-485-5p has recently been investigated in the serum of patients with prostate cancer and colorectal cancer patients. It was concluded that miR-485-5p serum levels could serve as diagnostic and prognostic biomarkers for these cancers [27, 28]. Serum levels of miRNA-485-5p were also found to be lower in acute myeloid leukemia patients than in healthy controls [37]. However, no reports were found about the pattern of circulating miR-485-5p in patients with HCC.

We explored the promising role of serum miR-485-5p levels in diagnosis and predicting prognosis in HCC related to HCV infection being the most common etiology in Egypt [32]. In our study, miR-485-5p was significantly downregulated in HCC patients. Its circulating values showed a stepwise decline pattern from the control group to cirrhotic patients, with the HCC group exhibiting the lowest levels. It was reported that miRNA-485-5p expression has been found to be downregulated in several tumor cells [38–40], including HCC tissue [21–23]. Previous reports indicated that miR-485-5p controls tumor growth as a tumor suppressor, which could explain the link between its low expression and the development and progression of tumors. Several mechanisms have been proposed for the involvement of miR-485-5p in HCC. An earlier study demonstrated that miR-485-5p inhibited HCC progression by downregulating the oncogene EMMPRIN [21].

Another study found that a direct target of miR-485-5p was stanniocalcin 2 (Stc2), a protein upregulated in HCC that predicts poor prognosis and promotes cell proliferation and migration. They showed that miR-485-5p could interact with the 3’-UTR of the Stc2 gene to inhibit its protein expression [22]. Furthermore, miR-485-5p was found to have a regulatory effect on the function of Mucin 1 (MUC1). This transmembrane glycoprotein participates in a variety of signal transduction pathways involving Bcl-2-associated X-protein (BAX), c-Jun N-terminal kinase/transforming growth factor beta (TGF-β), p53, nuclear factor-κB, and mitogen-activated protein kinase-extracellular-signal-regulated kinase (ERK)/ERK (MEK-ERK). MUC1 levels in HCC cells, which are modulated by miR-485-5p, might play a role in the HCC pathophysiological process by interfering with these signaling pathways [41]. In addition, it was reported that miR-485-5p directly targets the Frizzled-7 (FZD7) receptor to inhibit the Wnt/β-catenin signal pathway with negative regulation of both cytoplasmic and nuclear β-catenin, and the expression of Wnt/β-catenin pathway downstream targets c-Myc and cyclin D1 in HCC cells [42]. Moreover, miR-485-5p has been shown to downregulate WW domain binding protein 2 (WBP2) to inhibit the development of HCC by the blockade of the Wnt/β-catenin pathway [23].

We compared the diagnostic performance of miR-485-5p in discriminating HCC patients from those with liver cirrhosis using the ROC curve. We found that miR-485-5p outperformed AFP with a greater AUC (0.921 vs. 0.704) and higher sensitivity and specificity (92.0 and 84.0 vs. 64.0 and 60.0, respectively). These results indicate that aberrant expression of circulating miR-485-5p may help in early detection of HCC with high accuracy.

We also examined the correlation between miR-485-5p and clinical features of HCC patients. Lower levels of miR-483-5p were substantially associated with more advanced cirrhosis (Child–Turcotte–Pugh class C) and with multiple focal lesions. Additionally, there was a significant positive correlation between miR-483-5p and serum albumin as well as platelet count and a significant negative correlation with ALP, GGT, total bilirubin, and the size of the focal lesions. This suggests that serum miR-485-5p may be useful as a good prognostic marker in HCC patients. Furthermore, miR-485-5p demonstrated high discriminative ability to differentiate early BCLC stages with expected survival more than 5 years from late BCLC stages with expected survival less than 5 years. It showed higher AUC, sensitivity, and specificity (0.872, 85.19, and 82.61, respectively) than AFP (0.695, 62.96, and 60.87, respectively) in predicting the prognosis of patients with HCC. In accordance with our results, a low level of circulating miR-485-5p was able to differentiate patients with colorectal cancer from healthy subjects and could be used as a potential diagnostic biomarker. Moreover, it was significantly correlated with tumor stage as well as shorter overall survival [27]. Also, serum miR-485-5p negatively correlated with prostate cancer progression with decreased values in late stages and also has the potential for predicting postoperative recurrence [28].

## Limitations

Although the National Liver Institute, Menoufia University, is a tertiary referral center where patients were referred from different parts of our country, one of the current study's limitations was that it was limited to one center. Recruitment of participants from different backgrounds and demographics is warranted to ensure the reliability and generalizability of the results. Also, our study focused on miR-485-5p in comparison with AFP only. A comparative analysis is still needed with other new biomarkers like GALAD, des-γ-carboxy prothrombin, or glypican-3.

Furthermore, we examined the potential role of miR-485-5p in predicting patients' prognosis based on their expected survival according to the BCLC staging. Longitudinal studies considering additional HCC patients monitoring to detect the outcome after treatment together with survival analysis are required to confirm the prognostic role of miR-485-5p. It is critical to recognize these limitations because they underline the necessity of conducting long-term, multicenter studies on a large population and offer priceless insights for subsequent research.

## Conclusions

Circulating miR-485-5p can be a helpful novel noninvasive diagnostic and prognostic biomarker for the early detection and prediction of prognosis in patients with HCV-linked HCC.

## Supplementary Information

Below is the link to the electronic supplementary material.

Supplementary file1 (DOCX 22 kb)

## Data Availability

No datasets were generated or analyzed during the current study.

## Abbreviations

HCV: Hepatitis C virus
HCC: Hepatocellular carcinoma
BCLC: Barcelona clinic liver cancer
qRT-PCR: Quantitative real-time polymerase chain reaction
AUC: Area under the ROC curve
AFP: Alpha-fetoprotein
*p*: *p* Value
lncRNA: Long noncoding RNA
miRNA: MicroRNA
ELISA: Enzyme-linked immunosorbent assay
EDTA: Ethylenediaminetetraacetic acid
CBC: Complete blood count
ROC: Receiver operating characteristic curve
ALP: Alkaline phosphatase
GGT: Gamma-glutamyl transferase
Stc2: Stanniocalcin 2
WBP2: WW domain binding protein 2
