# Supplementary material for: Circulating miR-485-5p as a potential diagnostic and prognostic biomarker for HCV-related hepatocellular carcinoma
Source: Clin Exp Med. 2025 Apr 10;25(1):110. doi: 10.1007/s10238-025-01625-y (PMC11985578; doi:10.1007/s10238-025-01625-y)
Supplement: Supplementary file 1 — Supplementary file1 (DOCX 22 kb) [file 10238_2025_1625_MOESM1_ESM.docx]

**Supplementary table 1: Correlation between AFP and miR-485-5p with different parameters in HCC group**

|  | **AFP ng/mL** | | **miR-485-5p** | |
| --- | --- | --- | --- | --- |
|  | **r_s_** | **p** | **r_s_** | **p** |
| **miR-485-5p** | -0.667 | **<0.001*** | - | - |
| **Age** | -0.222 | 0.122 | 0.148 | 0.306 |
| **AST** | -0.148 | 0.306 | 0.064 | 0.658 |
| **ALT** | -0.076 | 0.601 | 0.182 | 0.206 |
| **ALP** | 0.061 | 0.674 | -0.430 | **0.002*** |
| **GGT** | 0.253 | 0.077 | -0.374 | **0.007*** |
| **Total bilirubin** | 0.171 | 0.234 | -0.454 | **0.001*** |
| **Direct bilirubin** | 0.038 | 0.794 | -0.073 | 0.615 |
| **Albumin** | 0.028 | 0.849 | 0.407 | 0.003 |
| **Creatinine** | 0.195 | 0.175 | -0.043 | 0.768 |
| **RBS** | -0.101 | 0.486 | 0.031 | 0.831 |
| **Hemoglobin** | -0.096 | 0.506 | 0.092 | 0.524 |
| **WBCs** | 0.009 | 0.951 | -0.122 | 0.397 |
| **Platelets** | 0.170 | 0.237 | 0.367 | **0.009*** |
| **INR** | 0.173 | 0.230 | -0.273 | 0.055 |
| **Hepatic focal lesion size** | 0.219 | 0.126 | -0.434^*^ | **0.002*** |

AFP: alpha-fetoprotein, AST: Aspartate transaminase, ALT: Alanine transaminase, ALP: Alkaline phosphatase, GGT: Gamma-glutamyl transferase, RBS: Random blood sugar, WBCs: White blood cells, INR: International normalized ratio, rs: Spearman coefficient, p: p value. *Statistically significant.

**Supplementary table 2: Diagnostic and prognostic performance of AFP and miR-485-5p**

|  | **AUC** | **p** | **95% C.I** | **Cut off** | **Sensitivity** | **Specificity** | **PPV** | **NPV** |
| --- | --- | --- | --- | --- | --- | --- | --- | --- |
| **Discriminate HCC patients (n = 50) from liver cirrhosis (n = 50)** | | | |  |  |  |  |  |
| - **AFP (ng/mL)** | 0.704 | **<0.001*** | 0.601 – 0.806 | >40 | 64.0 | 60.0 | 61.5 | 62.5 |
| - **miR-485-5p** | 0.921 | **<0.001*** | 0.868 – 0.973 | ≤0.9 | 92.0 | 84.0 | 85.2 | 91.3 |
| - **AFP + miR-485-5p** | 0.920 | **<0.001*** | 0.870 – 0.971 |  | 90.0 | 80.0 | 81.8 | 88.9 |
| **Discriminate late BCLC stage (n = 27) from early BCLC stage (n = 23) in HCC patients** | | | | | | | | |
| - **AFP( ng/mL)** | 0.695 | **0.019*** | 0.550 – 0.839 | >50 | 62.96 | 60.87 | 65.4 | 58.3 |
| - **miR-485-5p** | 0.872 | **<0.001*** | 0.765 – 0.979 | ≤0.51 | 85.19 | 82.61 | 85.2 | 82.6 |

HCC: hepatocellular carcinoma, BCLC: Barcelona clinic liver cancer, n: Number, AFP: Alpha-fetoprotein, AUC: Area Under Curve, p: p value, CI: Confidence Interval, PPV: Positive predictive value, NPV: Negative predictive value. *Statistically significant.
